# Antidepressant dosage taken by patients with bipolar disorder: factors associated with irregularity

**DOI:** 10.1186/2194-7511-1-26

**Published:** 2013-12-09

**Authors:** Rita Bauer, Tasha Glenn, Martin Alda, Kemal Sagduyu, Wendy Marsh, Paul Grof, Rodrigo Munoz, Greg Murray, Philipp Ritter, Ute Lewitzka, Emanuel Severus, Peter C Whybrow, Michael Bauer

**Affiliations:** Department of Psychiatry and Psychotherapy, Medical Faculty, Technische Universität Dresden, Fetscherstr 74, Dresden, 01307 Germany; ChronoRecord Association Inc, Fullerton, CA 92834 USA; Department of Psychiatry, Dalhousie University, Halifax, NS B3H 4R2 Canada; Department of Psychiatry, University of Missouri Kansas City School of Medicine, Kansas City, MO 64110 USA; Department of Psychiatry, University of Massachusetts, Worcester, MA 01655 USA; Department of Psychiatry, University of Toronto, Toronto, ON M5T 1R8 Canada; Mood Disorders Center of Ottawa, Ottawa, K1G 4G3 Canada; Department of Psychiatry, University of California San Diego, San Diego, CA 92093 USA; Faculty of Life and Social Sciences, Swinburne University of Technology, Melbourne, Victoria 3122 Australia; Department of Psychiatry and Biobehavioral Sciences, Semel Institute for Neuroscience and Human Behavior, University of California Los Angeles (UCLA), Los Angeles, CA 90095 USA

## Abstract

**Background:**

This study analyzed regularity in the daily dosage of antidepressants taken by patients with bipolar disorder and identified the factors associated with irregularity.

**Methods:**

Daily self-reported medication dosage taken and mood ratings were available from 144 patients who received treatment as usual. All 144 patients took the same antidepressant for at least 100 days. One hundred eleven of these patients were also taking a mood stabilizer. Approximate entropy (ApEn) was used to measure serial regularity in daily dosage. Regularity is the tendency that values within a time series remain the same on incremental comparisons. Drug holidays (missing three or more consecutive days) were also determined. Generalized estimating equations (GEE) were used to estimate if any demographic or clinical variables were associated with regularity.

**Results:**

Although the mean percent of days missing doses was only 18.6%, there was a wide range of regularity in the daily antidepressant dosage. Drug holidays were common, occurring in 41% of the analyses. Factors significantly associated with irregularity were as follows: total number of psychotropic medications (*p* = 0.005), pill burden (*p* = 0.005), and depression (*p* = 0.015). Neither the percent of days missing doses nor the drug holidays were associated with any demographic or clinical factors. For patients taking both antidepressants and mood stabilizers, there was no significant difference in regularity in daily dosage between these drugs.

**Discussion:**

There can be considerable irregularity in daily dosage despite a low percent of days missing doses. Medication regimen complexity and depressed mood are associated with increased irregularity. Daily regularity in drug dosage may be more dependent on the individual than on the specific drug. Research on the clinical impact of irregularity in daily dosage of antidepressants is needed.

## Background

Patients with bipolar disorder are expected to take many drugs for long periods of time. Polypharmacy is prescribed to about two thirds of patients with bipolar disorder in the USA and one half of those in Europe (Baldessarini et al. 2008a; Bauer et al. 2013a; Goldberg et al. 2009; Hayes et al. 2011; Quante et al. 2010). On entry to the STEP-BD study, 40% of the 4,035 patients were receiving three or more psychotropic drugs (Goldberg et al. 2009). The drugs prescribed most frequently for bipolar disorder are mood stabilizers and antidepressants (Baldessarini et al. 2006; Baldessarini et al. 2008a; Bauer et al. 2013a; Greil et al. 2012; Hayes et al. 2011; Haeberle et al. 2012). Despite ongoing disagreement as to the role of antidepressants in the treatment of bipolar disorder (Altshuler et al. 2003; Ghaemi et al. 2008; Möller and Grunze 2000; Pacchiarotti et al. 2013), long-term prescribing of antidepressants remains widespread in clinical practice. In recent international studies, about half the patients were prescribed antidepressants either as part of a polypharmacy regimen or as monotherapy (Baldessarini et al. 2006; Baldessarini et al. 2008a; Haeberle et al. 2012; Quante et al. 2010; Sussman et al. 2012).

Nonadherence with prescribed medication regimens remains a problem in almost half of patients with chronic illness regardless of specific diagnosis or drug (Briesacher et al. 2008; Haynes et al. 2008). About 40% of patients with bipolar disorder do not follow instructions for taking their medications, with most having intermittent or partial adherence with the prescribed dosing regimen (Lingram and Scott Lingam and Scott 2002). Both the therapeutic and side effects of a drug depend on the dosage strength and the dosing interval, and deviation from the prescribed regimen may result in a poor medication response (Urquhart 1996; Urquhart 1997). We previously found considerable irregularity in the daily dosage of mood stabilizers taken by patients who only failed to take medication on 14% of days (Bauer et al. 2013b). Regularity, as measured using approximate entropy (ApEn), is the tendency that values within a time series remain the same on incremental comparisons (Pincus et al. 1991). The purpose of this investigation was to analyze the regularity in the daily antidepressant dosage taken by patients with bipolar disorder based on self-reported data, and to evaluate factors that may influence regularity.

## Methods

All data were obtained from an ongoing, long-term naturalistic study in which patients with bipolar disorder recorded mood, sleep, and medications taken daily (Bauer et al. 2012). Patients were aged 18 years or older, diagnosed with bipolar disorder using DSM-IV criteria, and agreed to record mood, sleep, and medications daily using ChronoRecord software on a home computer for 6 months. The diagnosis was made by the prescribing psychiatrist at a clinical interview. Throughout the study, all patients remained under the care of a psychiatrist and received pharmacologic treatment as usual. All participants were volunteers who did not receive payment and had access to a home computer. All provided written informed consent, approved by the local Institutional Review Board, prior to the study.

### Data collection

All data were collected using the previously validated ChronoRecord software in the patient's native language (Bauer et al. 2004; Bauer et al. 2008). The participants entered a daily mood rating using a 100-unit visual analog scale that was calibrated to the extremes of mania and depression that the patient ever experienced. The daily self-ratings of hypomania and mania reflect activation levels for either euphoric or dysphoric mood (Bauer et al. 2004). Based on the validation studies, a mood entry of less than 40 was considered depression, 40 to 60 euthymia, and >60 hypomania/mania. Every day, the patients also recorded their sleep, psychotropic medications taken, and any significant life events.

The patient's bipolar disorder medications were entered during ChronoRecord training by selecting from a list in the software. The list displays the psychotropic medications for each country by brand and generic name, including antidepressants, mood stabilizers (lithium, valproate, lamotrigine, carbamazepine, or oxcarbazepine), antipsychotics, benzodiazepines, insomnia medications, other anticonvulsants, thyroid hormones, and estrogens. If there was a prescription change, the patient could modify the drugs taken and could add a drug that was not included in the software list. For each medication, the patient entered the total number of pills taken daily. Partial pills (1/4, 1/2, or 3/4) could be entered for tablets, but not for capsules. The patient entered a 0 if no pills were taken, and missing days of data were treated as no pills taken. The software includes error checking steps such as preventing entry of data for a future date and requiring confirmation for the entry of a large number of pills.

### Regularity analysis

The analysis of regularity in daily medication dosage was described previously (Bauer et al. 2013b) and is summarized here. ApEn computes a single, non-negative number, where 0 indicates a completely regular sequence and increasing positive values indicate increasing levels of irregularity (Pincus et al. 1991; Pincus et al. 1999). The estimated value of the ApEn (*m*,*r*,*N*) depends on *m* the pattern length used for prediction of the subsequent value, *r* the level of noise filtering, and *N* the number of dosage values in the run to be compared. The level of noise filtering was calculated as a percent of the individual subjects' standard deviation. The ApEn parameters *m* = 1 day, *r* = 0.2 × SD in daily antidepressant or mood stabilizer dosage, and *N* = 100 days were used in this analysis. The same data length was used for each ApEn analysis (Pincus et al. 1999). The value of ApEn is dependent on the order of data in a time series and changing the order of the data will likely change the ApEn. In contrast, the traditional mean and standard deviation will be identical for a set of values regardless of the order of the data used in the calculation. ApEn is most useful with partial adherence since the result would be 0 if a patient discontinued treatment or made no changes and is not largely affected by a prescription change if the new dosage is maintained (Bauer et al. 2013b).

### Data

For each patient, for each antidepressant, the time span for taking each antidepressant was determined. If the time span was ≥ 100 days, the ApEn was calculated for the first 100 days of data using the daily antidepressant dosage taken. The database contained 109,287 days of data from 475 patients who returned ≥ 30 days of data. Starting with 475 patients, 244 (51.4%) of the patients took an antidepressant for at least one day. Of these 244 patients, 184 (75.4%) were female, and 144 patients took antidepressants for ≥ 100 days and were included in the analysis. More than one ApEn analysis was completed if patients took more than one antidepressant for ≥ 100 days. Thirty-six of the 144 patients were not included in our prior analysis of regularity in daily mood stabilizer dosage (Bauer et al. 2013b). Of the 144 patients, 111 were taking at least one antidepressant and at least one mood stabilizer for ≥ 100 days. For these 111 patients, the ApEn analysis of the daily mood stabilizer dosage was also calculated.

### Statistical analysis

The demographic and clinical characteristics as measured by the mood ratings and psychotropic medications taken by the 144 patients were calculated. For each patient, the percent of days with euthymic, depressed, and hypomanic/manic mood were determined for each 100-day ApEn analysis period. For each patient, the mode of the daily number of medications and the number of pills (pill burden) for all psychotropic medications during the 100-day span were calculated. Also, for each patient, the mode of the daily antidepressant dosage was calculated for each patient for each antidepressant taken in the 100-day span. The mode is the most frequent value in a series of numbers and was chosen as a proxy for the prescribed daily number of medications, pill burden, and daily dosage. A generalized estimating equation (GEE) approach was used to adjust model coefficients and standard errors for within-patient correlation since a patient could take more than one antidepressant or mood stabilizer. To estimate if any demographic or clinical variables were associated with ApEn for antidepressants, GEE models used ApEn as the dependent variable with an independent working correlation structure (Pan and Connett 2002). GEE models were also used to estimate if demographic or clinical variables were associated with the percent of days missing doses, or with taking drug holidays. For patients taking both antidepressants and mood stabilizers, GEE models were used to estimate if the ApEn was significantly different between these drugs. SPSS 20.0 (Armonk, NY, USA) was used for all calculations.

## Results

The demographic characteristics of the 144 patients are shown in Table 1. The 144 patients took between one and four antidepressants: 96 took one antidepressant, 39 took two antidepressants, 3 took three antidepressants, and 6 took four antidepressants for a total of 207 ApEn analyses. The 144 patients returned a mean of 390.5 ± 194 days of data. During the 207 100-day analysis periods, the 144 patients were depressed on average for 23.8% of days, euthymic for 69.5% of days, and hypomanic/manic for 6.7% of days.

**Table 1:** **Patient demographics (** ***N*** **= 144)**

Demographics	Value
Gender, *n* (%)	
Male	36 (25.0)
Female	108 (75.0)
Diagnosis, *n* (%)	
BP I	67 (47.2)
BP II	65 (45.8)
BP NOS	10 (7.0)
Marital status, *n* (%)	
Married	70 (51.5)
Divorced	22 (16.2)
Single	44 (32.4)
Disabled, *n* (%)	
Yes	34 (26.0)
No	97 (74.0)
Education, *n* (%)	
High school	18 (13.3)
Some college	41 (30.4)
College graduate	76 (56.3)
Age, *n*; mean ± SD	144; 42.9 ± 11.2
Age of onset, *n*; mean ± SD	135; 23.1 ± 11.6
Hospitalizations, *n*; mean ± SD	133; 2.6 ± 4.0
Years of illness, *n*; mean ± SD	135; 20.3 ± 12.3

### Medication overview

The 144 patients took a mean of 4.4 ± 2.1 psychotropic medications daily with a mean pill burden of 7.6 ± 5.3. The psychotropic drugs taken by the 144 patients are shown in Table 2. The 144 patients took 20 different antidepressants, of which 8 were included in ≥ 10 analysis periods: bupropion in 40, venlafaxine in 28, escitalopram in 24, citalopram in 17, sertraline in 14, duloxetine in 13, paroxetine in 13, and fluoxetine in 11. During the 207 analysis periods, the mean percent of days missing doses of antidepressant was 18.6% ± 22.0%. There was no association between the percent of days missing doses of antidepressant and the pill burden (*p* = 0.396), total number of medications (*p* = 0.086), percent of days depressed (*p* = 0.070), percent of days euthymic (*p* = 0.135), or the percent of days manic (*p* = 0.839).

**Table 2:** **Patient medications (** ***N*** **= 144)**

	Value
Number of antidepressants, *n* (%)	
1	96 (66.7)
2	39 (27.1)
3	3 (2.1)
4	6 (4.2)
Taking mood stabilizer, *n* (%)	106 (73.6)
Taking benzodiazepine, *n* (%)	33 (22.9)
Taking antipsychotic, *n* (%)	57 (39.6)
Total pill burden^a^, mean ± SD	7.6 ± 5.3
Total number of medications^a^, mean ± SD	4.4 ± 2.1
Antidepressants^c^, mean days^b^; mean dosage mg ± SD	
Bupropion^c^	86; 277.3 ± 30.3
Citalopram	92; 37.4 ± 4.6
Duloxetine	77; 58.8 ± 6.0
Escitalopram	90; 15.3 ± 2.2
Paroxetine	87; 20.1 ± 4.1
Fluoxetine	90; 31.7 ± 6.6
Sertraline	85; 89.2 ± 9.1
Venlafaxine	82; 173.8 ± 11.9

^a^Psychiatric medications only. Calculated as mode for each patient.

^b^Excluding missing days.

^c^Only antidepressants included in ≥10 analysis periods.

**Figure 1:**
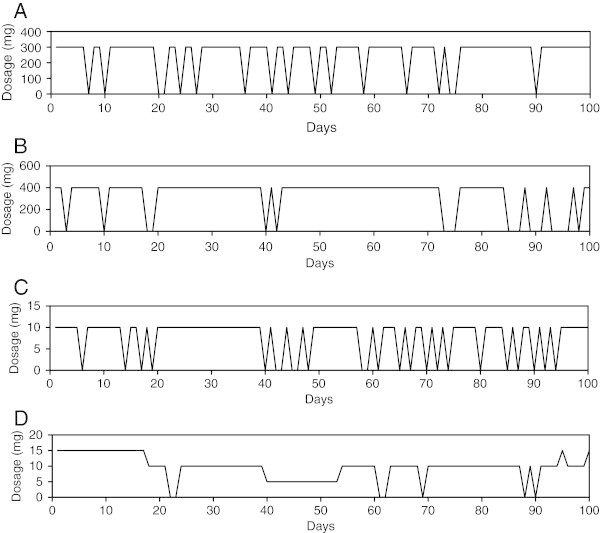
**Irregularity in antidepressant usage (high ApEn). (A)** Bupropion usage. ApEn(1,0.2 × SD,100) = 0.456, 17% missing days, 0 holidays. **(B)** Bupropion usage. ApEn(1,0.2 × SD,100) = 0.443, 20% missing days, 4 holidays. **(C)** Escitalopram usage. ApEn(1,0.2 × SD,100) = 0.541, 24% missing days, 0 holidays. **(D)** Escitalopram usage. ApEn(1,0.2 × SD,100) = 0.453, 7% missing days, 0 holidays.

There was at least 1 day of missing data in 136 (66%) of the 207 analysis periods. One or more drug holidays, defined as missing three or more consecutive days (Urquhart 1997), was present in 85 (41.1%) of the analysis periods. See Table 3. Of the 85 analysis periods, with at least one drug holiday, more than one drug holiday was present in 38 (44.7%). There was no significant association between drug holidays and any demographic or clinical values. Figure 1 provides examples of patients with irregular antidepressant dosage despite a low percent of days missing doses, related to the missing days and drug holidays.

**Table 3:** **Patient frequency of drug holidays (** ***N*** **= 207)**

Frequency^a^	***N*** (%)
0	122 (58.9)
1	47 (22.7)
2	18 (8.7)
3	14 (6.8)
4	5 (2.4)
5	1 (0.5)

^**a**^Drug holiday defined as missing three or more consecutive days.

### Regularity analysis

The 207 ApEn results for antidepressants ranged between 0 and 1.04, with a mean of 0.21 (SD 0.19), with 120 (58%) between 0 and 0.2, 55 (27%) > 0.2 and ≤0.4, and 32 (15%) > 0.4. Only 15% (*n* = 31) of the patients had an ApEn of 0, meaning no change in the daily antidepressant dose across the 100-day analysis period. As shown in Table 4, the total number of medications, the pill burden, and depression were associated with increased irregularity (larger ApEn), while euthymia was associated with decreased irregularity (smaller ApEn).

**Table 4:** **Estimated parameter coefficients and significance of 144 patients taking 207 antidepressants for 100 days** ^**a**^

Parameter	Coefficient estimate	95% Wald confidence interval	Wald chi-square	***P***
Total pill burden	0.009	(0.003, 0.015)	8.004	0.005*
Total number of medications	0.020	(0.006, 0.033)	7.857	0.005*
Percent days depressed	0.002	(<0.001, 0.003)	5.865	0.015*
Percent days euthymic	−0.001	(−0.003, <0.000)	3.891	0.049*
Percent days manic	0.000	(−0.004, 0.003)	0.065	0.799

^a^GEE model estimated ApEn (1,0.2 × SD,100) using listed parameters with an independent correlation structure for each patient. Degrees of freedom were 1 for all models.

*Significant < 0.05.

### Antidepressants and mood stabilizer analysis

The 111 patients who were taking both a mood stabilizer and an antidepressant took between one and four antidepressants: 72 took one antidepressant, 31 took two antidepressants, 2 took three antidepressants, and 6 took four antidepressants for a total of 164 ApEn analyses for antidepressants. The 111 also took between one and three mood stabilizers: 76 took one mood stabilizer, 32 took two mood stabilizers, and 3 took three mood stabilizers for a total of 149 ApEn analyses for mood stabilizers. In total, there were 313 ApEn analyses for those taking both mood stabilizers and antidepressants. For the 111 individuals, there was no significant difference in the regularity for taking an antidepressant versus taking a mood stabilizer (*p* = 0.273).

## Discussion

Across individuals, a wide range of irregularity in the daily dosage of antidepressants was found among patients who were motivated to record mood daily and who took medication on 81% of days. The irregularity was primarily due to single-day omissions and changes in daily dosage. Drug holidays were also common, with at least one drug holiday occurring in 41% of the analysis periods. The lack of association between the percent of days missing doses and either mood, number of daily medications or pill burden suggests that regularity and the percent of days missing doses are measuring different aspects of adherence (Bauer et al. 2013b). Regularity, as measured by ApEn, should be used in conjunction with summary statistics, as each provides separate information.

Irregularity in daily dosage is important since it may contribute to individual variation in drug response (Harter and Peck 1991; Urquhart 1997; Vrijens et al. 2005). In this study, depression, the pill burden, and total number of psychotropic medications were associated with increased irregularity in daily antidepressant dosage, while euthymia was associated with increased regularity. The same factors were associated with irregularity in our prior study of daily mood stabilizer dosage (Bauer et al. 2013b). Evidence from adherence research also supports these findings. Depression, including residual symptoms, was associated with nonadherence in bipolar disorder (Baldessarini et al. 2008b; Johnson et al. 2007; Belzeaux et al. 2013) and in many general medical conditions (DiMatteo et al. 2000). Several reports relate nonadherence with medication regimen complexity in bipolar disorder (Bauer et al. 2013b; Keck et al. 1996; Lavantes et al. 1999; Sajatovic et al. 2009), although research on this issue is limited. An association between medication regimen complexity and nonadherence was found across a wide range of chronic medical conditions (Ingersoll and Cohen 2008).

Medication regimen complexity is of growing concern in bipolar disorder for several reasons. The use of polypharmacy has increased sharply over the last decade (Mojtabai and Olfson 2010; Greil et al. 2012; Haeberle et al. 2012), and many patients must take complex regimens throughout their lifetime, even when asymptomatic. Although patients with bipolar disorder frequently have medical comorbidities (Kilbourne et al. 2004; Krishnan 2005), this study only considered psychotropic drugs, and regimens may be considerably more onerous when all drugs are included. Medication regimen complexity would also be expected to increase as patients age and experience more chronic diseases. In a nationally representative community sample of 3,005 adults over age 57 in the US, 29% used more than five prescription medications, and about half took over the counter preparations or supplements (Qato et al. 2008). Finally, an important characteristic of patients who are adherent to complex medication regimens is the ability to successfully integrate the dosing schedule into their daily routine (Ryan and Wagner 2003; Vrijens et al. 2005). Yet many patients with bipolar disorder have an unstable lifestyle and a chaotic daily routine (Frank et al. 2000).

When patients took both an antidepressant and a mood stabilizer, there was no difference in regularity in the daily dosage between the drugs. This suggests that regularity in daily dosage may be independent of the specific drug and depend primarily on the individual. This finding is consistent with previous work on adherence in patients receiving polypharmacy. Similar nonadherence rates were found for both antidepressants and mood stabilizers in patients with psychiatric disorders (Bulloch and Patten 2010; Colom et al. 2000), and for several classes of drugs in patients with bipolar disorder (Sajatovic et al. 2009). Furthermore, regardless of diagnosis, people with chronic illness often experiment with their medications. Many will alter medication dosage up or down as they perceive the symptoms are changing (Pound et al. 2005), or actively minimize intake as a form of asserting control over a difficult illness (Conrad 1985; Cooper et al. 2007; Pound et al. 2005).

It is not clear how much irregularity in daily dosage will still provide acceptable therapeutic coverage for the drug regimens in this study. The relationship between adherence and outcome is complex, as the pharmacodynamic and pharmacokinetic properties of the each drug and drug formulation directly impacts the effects of dosing irregularities. Forgiveness is defined as the difference between the postdose duration of beneficial action and the dosing interval, and varies with half life and dosage strength (Osterberg et al. 2010; Urquhart 1998). With more forgiving drugs, drug action may continue when a dose is missed. To address the problem of missed doses, many newer drug formulations require less frequent dosing, often once daily. However, the consequences of missing a dose of a drug with less frequent administration may be more severe than for more frequent administration (Comté et al. 2007; Osterberg et al. 2010). While less frequent dosing does increase adherence (Claxton et al. 2001; Saini et al. 2009), this often does not lead to improved outcomes for patients with chronic diseases (Richter et al. 2003). Future studies of the impact of the frequency of dosing of antidepressants on the outcome in bipolar disorder are indicated.

The frequency of drug holidays in the short 100-day analysis periods are of particular concern since abrupt discontinuation of all classes of antidepressants, and especially short-acting serotonin uptake inhibitors, may trigger withdrawal reactions (Haddad 2001; Judge et al. 2002; Rosenbaum et al. 1998). In prior studies of patients with depression, symptoms were milder after abrupt discontinuation of fluoxetine with a long half-life of 2 to 6 days than after abrupt discontinuation of paroxetine with a half-life of 21 h (Judge et al. 2002; Osterberg et al. 2010; Rosenbaum et al. 1998). In addition to immediate symptoms related to drug half-lives, late-appearing symptoms related to long-term adaptive responses to cerebral pharmacodynamic effects may also occur (Baldessarini 1995; Baldessarini et al. 2010; Osterberg et al. 2010). Moreover, rapid discontinuation of an antidepressant, rather than gradual tapering, is associated with a shorter time to depression recurrence in patients with bipolar disorder (Baldessarini et al. 2010). For patients taking antidepressants, investigation of the impact of drug holidays on the course of bipolar disorder is needed.

This study may underestimate the irregularity in the daily antidepressant dosage for several reasons. Dosage timing was not investigated, as would be possible with an electronic medication monitor. Since the analysis required 100 days of data, the least adherent patients were excluded. Although patients in the complete ChronoRecord database have similar demographic characteristics to those who participate in other large studies of bipolar disorder (Bauer et al. 2012), only a subset was included in this analysis. The subset was predominantly female, educated, and only failed to take medication on 19% of days. While the findings may not be generalizable to all patients with bipolar disorder, even higher irregularity in the daily antidepressant dosage would be expected in patients who are less adherent.

There were other limitations to this study. All data were self-reported. The prescribed dosage for the antidepressant was not known, although the mean daily dose of each antidepressant was in the expected range. Other factors that impact medication regimen complexity such as instructions to take drugs separately or with food were not considered (Libby et al. 2013). Diverse factors that contribute to adherence were not included in this study such as attitudes towards bipolar disorder, psychiatric comorbidities, substance abuse and psychotic symptoms (Berk et al. 2010; Leclerc et al. 2013; Keck et al. 1998; Dell'Osso et al. 2002), out-of-pocket costs of prescription drugs (Piette et al. 2004), frequency of psychiatric visits (Patel et al. 2005), and the quality of patient-physician communication (Zolnierek and DiMatteo 2009). The ApEn technique does not address causality.

A strength of this study is that the analysis was based on the daily dose taken by the patient. Most instruments used to measure adherence in bipolar disorder focus on the number of missing days and the attitudes and behaviors associated with adherence, rather than on what was ingested (Berk et al. 2010; Sajatovic et al. 2010). Although self-reported instruments are subjective, review articles on adherence report moderate to high agreement between self-report and electronic medication monitoring devices (Garber et al. 2004; Shi et al. 2010). Additionally, good agreement between patient questionnaires and serum drug levels has been reported in bipolar disorder (Jónsdóttir et al. 2010; Lam et al. 2003).

## Conclusion

In conclusion, considerable irregularity was found in the daily dosage of antidepressants taken despite a low percent of days missing doses. Drug holidays were common. Depression, the number of daily pills, and the pill burden were associated with increased irregularity, and euthymia was associated with regularity. For patients who took both antidepressants and mood stabilizers, there was no significant difference in the regularity in daily dosage between these drugs. Further research is required to identify medication regimens for bipolar disorder that are more suitable for imperfect adherence.
